# Supplementary material for: Changes in visual acuity and retinal microstructures following vitrectomy for lamellar macular hole
Source: PLoS One. 2026 Feb 11;21(2):e0342652. doi: 10.1371/journal.pone.0342652 (PMC12893583; doi:10.1371/journal.pone.0342652)
Supplement: S1 Table — (PDF) [file pone.0342652.s001.pdf]

	Post-BCVA (BSS)	Post-BCVA (Air)	p-value
**Preoperative**	0.27 ± 0.20	0.39 ± 0.36	0.382
**Month 1**	0.80 ± 0.26	0.70 ± 0.31	0.313
**Month 3**	0.86 ± 0.25	0.73 ± 0.36	0.311
**Month 6**	0.91 ± 0.25	0.78 ± 0.33	0.351

Mann–Whitney U test
