# Supplementary material for: Changes in visual acuity and retinal microstructures following vitrectomy for lamellar macular hole
Source: PLoS One. 2026 Feb 11;21(2):e0342652. doi: 10.1371/journal.pone.0342652 (PMC12893583; doi:10.1371/journal.pone.0342652)
Supplement: S2 Table — (PDF) [file pone.0342652.s002.pdf]

			BSS	Air	p-value
**Preoperative**	Presence of inner retinal cyst	(+) n (%)	1 (8.3%)	8 (26.7%)	0.247
		(-) n (%)	11(91.7%)	22 (73.3%)	
	Presence of outer retinal cyst	(+) n (%)	4 (33.3%)	19 (63.3%)	0.098
		(-) n (%)	8 (66.7%)	11 (36.7%)	
	EZ disruption	(+) n (%)	5 (41.7%)	17 (56.7%)	0.499
		(-) n (%)	7 (58.3%)	13 (43.3%)	
	Presence of EP	(+) n (%)	5 (41.7%)	22 (73.3%)	0.078
		(-) n (%)	7 (58.3%)	8 (26.7%)	
**Month 1**	Presence of inner retinal cyst	(+) n (%)	1(9.1%)	5(17.2%)	1
		(-) n (%)	10(90.9%)	24(82.8%)	
	Presence of outer retinal cyst	(+) n (%)	2(18.2%)	14(48.3%)	0.148
		(-) n (%)	9(81.8%)	15(51.7%)	
	EZ disruption	(+) n (%)	3(27.3%)	17(58.6%)	0.155
		(-) n (%)	8(72.7%)	12(41.4%)	
**Month 3**	Presence of inner retinal cyst	(+) n (%)	0(0%)	4(27.6%)	0.554
		(-) n (%)	9(100%)	25(72.4%)	
	Presence of outer retinal cyst	(+) n (%)	2(22.2%)	8(27.6%)	1
		(-) n (%)	7(77.8%)	21(72.4%)	
	EZ disruption	(+) n (%)	3(33.3%)	16(55.2%)	0.447
		(-) n (%)	6(66.7%)	13(44.8%)	
**Month 6**	Presence of inner retinal cyst	(+) n (%)	1(33.3%)	1(6.3%)	1
		(-) n (%)	8(66.7%)	15(93.7%)	

Presence of outer retinal cyst	(+) n (%)	1(33.3%)	2(12.5%)	1
	(-) n (%)	8(66.7%)	14(87.5%)	
EZ disruption	(+) n (%)	3(33.3%)	7(43.8%)	0.691
	(-) n (%)	6(66.7%)	9(56.2%)	

Fisher's exact test.
